# Robust Curb Detection with Fusion of 3D-Lidar and Camera Data

**DOI:** 10.3390/s140509046

**Published:** 2014-05-21

**Authors:** Jun Tan, Jian Li, Xiangjing An, Hangen He

**Affiliations:** 1 College of Mechatronic Engineering and Automation, National University of Defense Technology, Changsha 410073, Hunan, China; E-Mails: lijian.nudt@gmail.com (J.L.); anxj@nudt.edu.cn (X.A.); hehangen@yahoo.com (H.H.); 2 National University of Singapore, Singapore 117583, Singapore; E-Mail: A0091500@nus.edu.sg

**Keywords:** curb detection, fusion, 3D-lidar, camera, depth image, Markov chain

## Abstract

Curb detection is an essential component of Autonomous Land Vehicles (ALV), especially important for safe driving in urban environments. In this paper, we propose a fusion-based curb detection method through exploiting 3D-Lidar and camera data. More specifically, we first fuse the sparse 3D-Lidar points and high-resolution camera images together to recover a dense depth image of the captured scene. Based on the recovered dense depth image, we propose a filter-based method to estimate the normal direction within the image. Then, by using the multi-scale normal patterns based on the curb's geometric property, curb point features fitting the patterns are detected in the normal image row by row. After that, we construct a Markov Chain to model the consistency of curb points which utilizes the continuous property of the curb, and thus the optimal curb path which links the curb points together can be efficiently estimated by dynamic programming. Finally, we perform post-processing operations to filter the outliers, parameterize the curbs and give the confidence scores on the detected curbs. Extensive evaluations clearly show that our proposed method can detect curbs with strong robustness at real-time speed for both static and dynamic scenes.

## 1. Introduction

Curb detection is a crucial component in both Autonomous Land Vehicles (ALV) and Advanced Driver Assistance Systems (ADAS). Robust curb detection in real environments can undoubtedly improve driving safety and benefit those systems in various tasks. In urban environments, curbs limit the driving area. They even have the same value as obstacles, for vehicles should not drive across the curbs. Curbs can also support map building and vehicle localization [1], with their continuous and static properties relative to the scenes.

**Figure 1.**
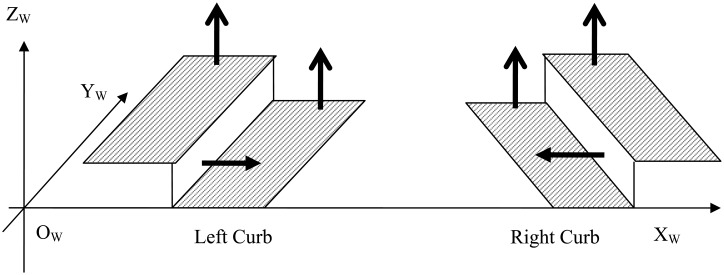
Geometric model of curbs. The short arrows indicate the surface normal directions in different positions.

Curbs are continuous objects in the road scene and thus they have specific geometric and visual properties which serve as the backbone for robust curb detection. Traditionally, these properties are captured separately by different sensors. More specifically, range sensors measure the geometric property, while visual sensors capture the visual appearance property. We now introduce the properties of curbs in details and then explain how to better use these properties in our method to detect the curbs. From the geometric model shown in Figure 1, we can observe following critical geometric properties of curbs:

1. *Height property*. There is a height variation over curbs, and the variation range is from 5 to 35 cm.
2. *Normal property*. The normal directions change sharply near curbs. More specifically, the normal directions around the left curb are ‘up-right-up’, while the ones around the right are ‘up-left-up’.
3. *Consistency property*. The above two properties are consistent and continuous along the curbs.

**Figure 2.**
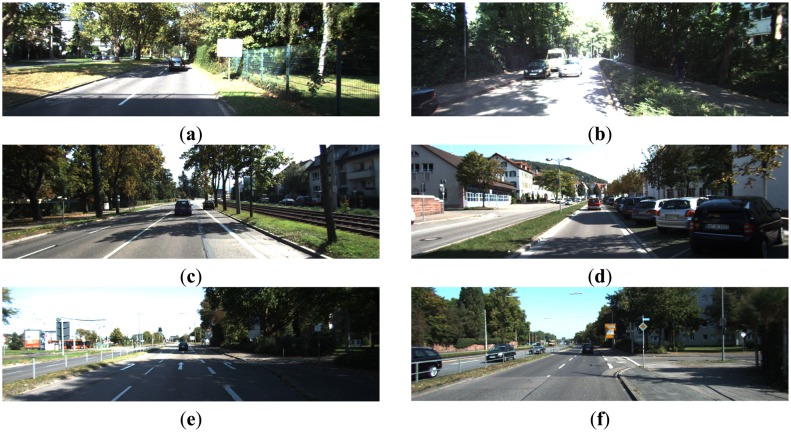
Curb images. (**a**) Curbs in illumination change; (**b**) Curbs in shadow; (**c**) Curbs in wide road; (**d**) Curbs in narrow road; (**e**) Curved curbs; (**f**) Long and short curbs.

From the road images containing curbs, as shown in Figure 2, we can observe the following visual properties of curbs:

1. *Edge property*. Some curbs have visible edges in the visual image;
2. *Variety property*. There is no universal appearance model of curbs applying for different scenes.

Though curbs have the aforementioned specific properties, curb detection in various environments remains a challenging problem, even with state-of-the-art sensors. The major difficulty lies in that the curbs only have a subtle variation of height, compared with obstacles. Such subtle range changes are easily confused with noises and are hardly detected by range sensors. On the other hand, for visual sensors, edges of the curbs are prone to being confused with other objects. Such cases will be worse in cluttered scenes.

Thus, range-visual fusion seems to be a promising approach for robust curb detection. Traditional range-visual fusion methods [2,3] used the detection results from range data to guide the curb searching in the visual image, which have the advantage of enlarging the detection range. However, curb searching in visual images may be unreliable in cluttered scenes, due to the visual properties mentioned above. Therefore, in this work, we propose an alternative approach to more effectively fuse the range and visual data to enhance the robustness of curb detection. In particular, we base our approach on the state-of-the-art range-visual fusion algorithm [4], which can recover a dense depth image of the scene by propagating depth information from sparse range points to the whole high-resolution visual image. With this high-quality recovered dense depth data, our method makes full use of the aforementioned geometric properties of the curbs for robustly detecting curbs in various conditions. Our proposed method roughly comprises following four steps.

Step 1: *Depth image recovery*. Provided with the sparse Lidar points and high-resolution camera images, we recover the dense depth image of the dynamic scene based on a filter-based fusion framework [4].
Step 2: *Curb point detection*. Based on the depth image representation, we propose a filter-based method for surface normal estimation. Using the normal property, curb point features are detected in the normal image row by row.
Step 3: *Curb point linking*. As aforementioned, curbs possess the consistency property. To describe this property, we construct a Markov Chain for each road side, where each curb point is represented as a node in the chain and the connection probabilities between nodes are computed based on the consistency degree of the neighboring nodes. The optimal curb paths which link the curb points are thus found by dynamic programming.
Step 4: *Curb refinement and parametrization*. For filtering out the outliers, break points are used to cut the obtained optimal curb path into multiple segments. We choose the segment with the maximum probability as our final result, parameterize the curb by using weighted least square fitting, and compute its confidence score by considering both the node probabilities and the accuracy of the model.

**Figure 3.**
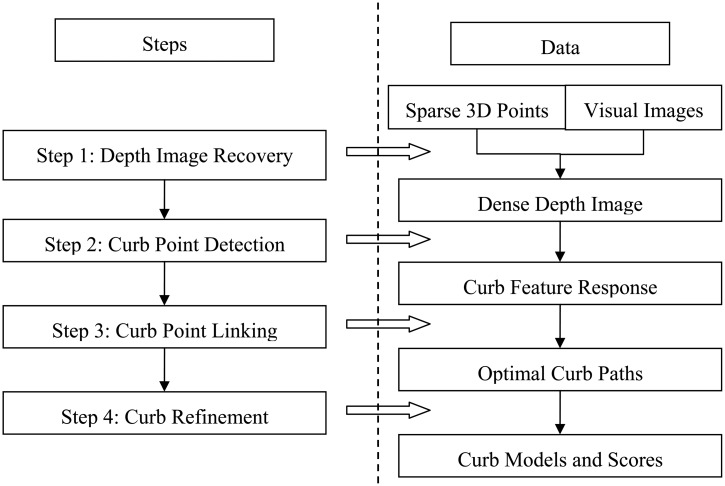
Overview of our proposed method. The method consists of four steps. For more details, please refer to the text.

A diagram illustrating the above four steps is given in Figure 3.

The major contributions of this paper can be briefly summarized as follows:

1. To the best of our knowledge, we are almost the first to use the dense depth image, which are obtained by effectively fusing the 3D-Lidar and camera data, for curb detection. The advantages of such data fusion for curb detection are well demonstrated in the experiments.
2. We propose a novel filter-based method for efficient surface normal estimation, and we show that the normal image can be used to accurately detect curb point features.
3. We build a Markov Chain model, using the curb point detection result, which elegantly captures the consistency property for curb point linking. The optimal curb path for each side is then linked by simple dynamic programming, which is computationally fast and cheap.
4. We propose several effective and feasible post-processing steps to filter out the outliers, to parameterize the curbs, and to obtain the confidence scores.
5. Based on the above proposed methods, we obtain robust curb detection results in various conditions (including quite bad conditions) efficiently within a long range, which is up to 30 m from the sensors. This is the best result ever achieved for curb detection to our best knowledge.

The remainder of the paper is organized as follows: in Section 2, we briefly review the existing curb detection methods with different sensors. In Section 3, we provide a detailed description of our proposed method for curb detection. In Section 4, comprehensive experiments are demonstrated in various scenes along with the quantitative and qualitative illustrations of our method. Finally, we give our conclusions and the future directions in Section 5.

## 2. Related Works

The research on curb detection has a long history [2], but it is still an attractive topic in intelligent robotics [5–7]. The development of these detection methods has generally followed improvements of the corresponding sensors. According to the used sensors, existing curb detection methods can mainly be categorized into: camera-based [6–13], Lidar-based [1,5,14–18], and fusion-based [2,3].

Camera-based curb detection methods have the advantage of low cost, but their performance is strongly sensitive to the outdoor conditions. For example, in low illumination, textureless, or cluttered scenes, their curb detection results may be unstable. Monocular methods utilize edge cues, and/or use texture information combined with machine learning techniques [7]. However, in curb detection, the edge cues can hardly be used as the curb edges are easy to be confused with other objects in cluttered scenes. The learning-based methods cannot apply for curb detection either. The reason is that the road and curb have great variations, and the model learned from one scene may not be suited for others. In [7], the authors tried to use Structure From Motion (SFM) to assist the information, but SFM cannot provide reliable structure estimation results under different conditions. Recently, stereo methods have become the most popular ones for curb detection [8–13]. These methods usually build the Digital Elevation Map (DEM) from the disparity, and use the edges with a certain height variation in the DEM as the cues for curb point detection. However, in textureless areas, stereo methods generally cannot provide stable range measurements, and this limits their applications.

In contrast with camera-based methods, Lidar-based methods can achieve reliable and accurate results in their valid range. However, common used Lidar sensors can only provide sparse data in a certain range, so their applicability is severely restricted in a limited area. For instance, a 2D-Lidar can only measure the distance in one scanning plane each time [5,14,15]. In order to improve the detection reliability, sequential data can be aligned with the ego-motion states [5]. At the same time, the error from ego pose estimation, which may come from abnormal shaking or relative pose drifting, will contaminate the detection result. A 3D-Lidar can give multilayer scanning data of the scene. The methods with the 3D-Lidar usually segment the ground plane first [19], and then detect the curbs in the ground surface [16,18]. For example, in [18] the authors used surface normal information, and the curbs were defined as the boundary of the plane in [17]. All those methods achieved remarkable results in their specific applications.

Fusion-based methods generally achieve better results, compared with pure camera or Lidar based methods, by integrating different information. The principle of existing fusion-based methods is to estimate reliable curbs in near region with range data at first, and then extend this result to be faraway by using the image data [2,3]. In [2], previous detection results were projected into the current image frame, and used to initialize the curb tracking in the image. In [3], the authors detected the curbs in stereo data, and used the results as the supervision signal to learn the monocular model for extended searching. The fusion-based curb tracking utilizing both 2D-lidar and camera data was proposed in [20]. There are still other sensors used for curb detection, such as the Time-of-Flight (ToF) camera [21]. However, this kind of sensor does not work at long range, or in bright sunlight.

No matter what sensors are used, all the above methods need a curb model. There are various models used in different methods, such as the straight line and line segment chain model [8], the polynomial model [9], and the polynomial spline model [10]. Generally, simple models are more robust to outliers, and sophisticated models are more accurate for describing the curb. The model parameters can be estimated by Hough Transform [8], RANdom SAmple Consensus (RANSAC) [9,18] or Weighted Least Square [2]. In [11,12] both the ground plane and curb were modeled, and in [12] past information was integrated, which can detect curbs in a range of 20 m

Other cues can also help to improve curb detection, such as the static property of the curb and the detection results of obstacles. Curbs are static relative to the road, hence, multi-frame data can be aligned to keep the persistent ones for denoising [8–10]. Explicit obstacle detection can also benefit curb detection, by removing the candidate points in obstacle region [2].

## 3. Fusion-Based Curb Detection Method

An overview of our method is provided in the Introduction. In this section, we introduce the details of four components of our proposed method along with the implementation details.

### 3.1. Depth Image Recovery

Geometric properties are important cues for curb detection. However, as mentioned, the geometric properties measured solely from the sparse range data are not robust and reliable for curb detection. In order to more effectively utilize the geometric properties of curbs, in this work, we propose to first recover a *dense* depth image of the scene for better curb detection, through fusing sparse range points (from the Lidar sensor) and high-resolution camera images. The dense depth image obtained in the fusion has the same resolution as the input visual images, and meanwhile has the precise depth value for each point/pixel. Thus the recovered depth image has significantly larger signal-to-noise ratio than the original range data, which is advantageous for the following curb detection. In particular, the fusion method used in this work is built on the observations that different points sharing similar features (including position, time, color, and/or texture *etc.*) generally have similar depth values. Fusion methods based on such assumption have shown great success in recent works [4]. Specifically, in this paper, we use the fusion algorithm proposed in [4] considering its real-time efficiency and outperforming accuracy in dynamic environments. In the implementation, the algorithm first aligns the sparse depth points with the points in the visual image frames. Then a high dimensional Gaussian filter is applied to spread the depth information from sparse points to all the image points at real time speed and generate the recovered dense depth image. For more details of the algorithm, we refer interested readers to the original paper [4].

**Figure 4.**
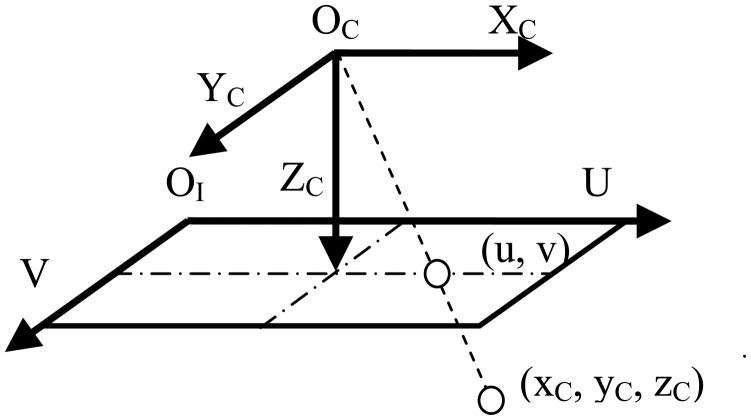
Illustration of a depth image within both the camera coordinate and image coordinate systems.

The recovered depth image is located within two coordinate systems, *i.e.*, the image coordinate system and the camera coordinate system, as shown in Figure 4. The image coordinate system is fixed with respect to the image frame, whose origin is located at the left-top corner of the image, U-axis direction is from left to right, and V-axis direction is from top to down. Different from the image coordinate system, the camera coordinate system is fixed with respect to the camera, with its origin located at the optical center and its X_C,_ Y_C,_ Z_C_–axis paralleling with U, V, optical-axis respectively. The units of image coordinates are in terms of pixels, while the units of camera coordinates are based on meters.

Figure 4 shows a depth image in these two coordinate systems. For a point having the camera coordinates (*x_C_*, *y_C_*, *z_C_*), the coordinates of its projection point in the image coordinate system (*u*, *v*) can be computed as in Equations (1) and (2) following the pinhole camera model:

(1) $$u=f_{x}\times \frac{x_{C}}{z_{C}}+c_{x}$$

(2) $$v=f_{y}\times \frac{y_{C}}{z_{C}}+c_{y}$$

In Equations (1) and (2), (*f_x_*, *f_y_*, *c_x_*, *c_y_*) are intrinsic camera parameters, which can be obtained in advance and known here. In this paper, we use the pinhole model without lens distortion to do the projection. In the cases that there is significant distortion, a distortion correction step should be conducted before the coordinate computations.

Let *D* denote a depth image, and *d*(*u*, *v*) denote the depth value of the point with image coordinates (*u*,*v*). As aforementioned, *D* has the same resolution as the input visual image. *d*(*u*, *v*) equals to the z_C_ in Equations (1) and (2), which is obtained from the above fusion method.

**Figure 5.**
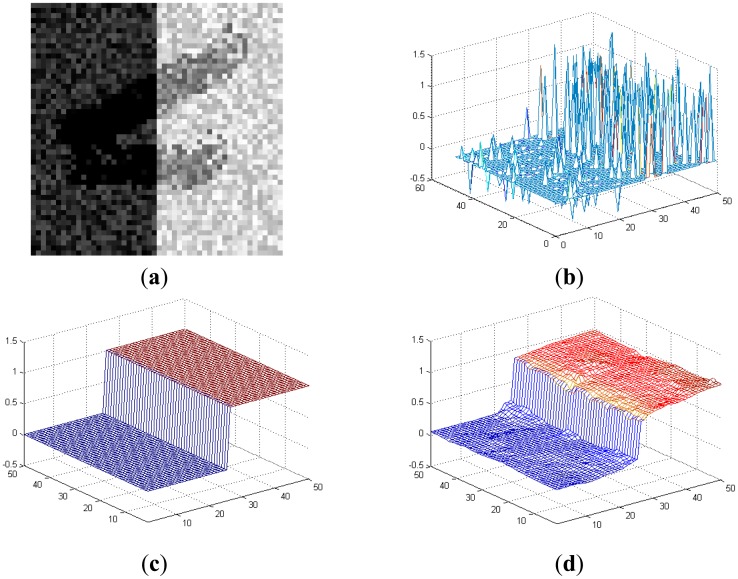
(**a**) Visual image containing noises and shadow; (**b**) Sparse range points; (**c**) Ground truth curb data; (**d**) Recovered depth image shown in 3D.

Here we provide an illustrating example in Figure 5 to demonstrate the advantages of using the depth image for curb detection. Figure 5a shows the top view of the visual image (containing random noises and shadow) of a curb segment, and Figure 5b shows the sparse range data aligned with the image. Due to the noises and shadow, we cannot accurately detect the curb on the visual or range data individually. Figure 5d shows the depth image recovered via fusing Figure 5a and Figure 5b. Obviously, recovering the dense depth image increases the resolution and meanwhile suppresses the noises/shadow of Figure 5a,b. Moreover, the curb geometric properties are clearly presented, compared with the ground truth curb shown in Figure 5c. This makes the curb detection and localization easier and more accurate.

**Figure 6.**
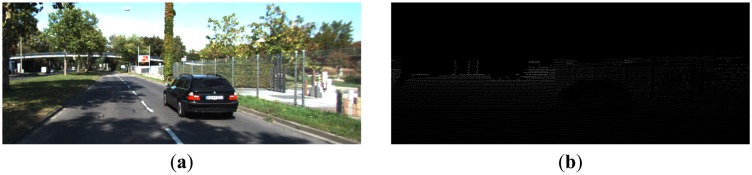

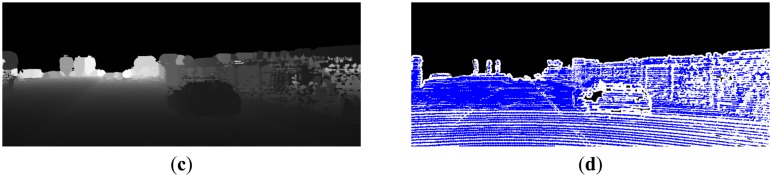
(**a**) Visual image; (**b**) Sparse range data in image frame; (**c**) Recovered depth image; (**d**) Confidence scores of the dense depth estimation.

A real example of depth recovery in outdoor scenes is demonstrated in Figure 6. Similar to the above example, we obtain a dense depth image of the scene along with its confidence using the algorithm in [4]. It can be observed that depth recovery also increases the resolution and filters out the noises for the realistic scene. The confidence scores are shown in Figure 6d, in which three colors (black, white, blue) are used to distinguish different confidence levels (zero, low, high), as [4].

**Figure 7.**
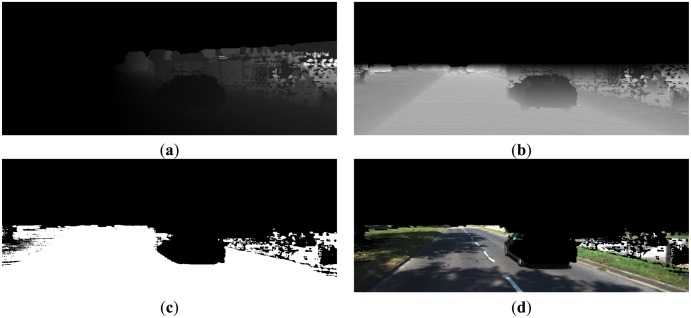
(**a**) X_C_ Image; (**b**) Y_C_ Image; (**c**) Road region; (**d**) Road image.

With the depth image, it is easy to calculate the word coordinates and camera coordinates for each point. The word coordinate system is fixed with respect to the vehicle, with its origin located at the projection point of the camera centre on the ground plane, its X_W_-axis pointing to the right side, Y_W_–axis pointing to ahead, and Z_W_–axis pointing to the up side. The extrinsic parameters translating world coordinates to camera coordinates are the rotation matrix R and the translation vector T, which can be estimated in advance and assumed to be known here. With *d*(*u*, *v*) and known intrinsic parameters, we estimate the camera coordinates(*x_C_*, *y_C_*, *z_C_*) as in Equations (3), (4) and (5). The X_C_, Y_C_ images are shown in Figure 7a,b, respectively. Then, by applying Equation (6), world coordinates can be readily obtained with known (*x_C_*, *y_C_*, *z_C_*) and extrinsic parameters:

(3) $$x_{C}(u,v)=\frac{u-c_{x}}{f_{x}}\times d(u,v)$$

(4) $$y_{C}(u,v)=\frac{v-c_{y}}{f_{y}}\times d(u,v)$$

(5) $$z_{C}(u,v)=d(u,v)$$

(6) $$\left[\begin{array}{c} x_{W}\\ y_{W}\\ z_{W} \end{array}\right]=R^{-1}•\left(\left[\begin{array}{c} x_{C}\\ y_{C}\\ z_{C} \end{array}\right]-T\right)$$

When z_W_ is known, it is easy to identify the road region, whose *z_W_* < *T_z_*. In this paper, we set *T_Z_* = 0.4 m, and the found road region and road image are shown in Figure 7c,d.

### 3.2. Curb Point Detection

After recovering the depth image and point coordinates in several systems, we now proceed to perform the curb point detection. First, we devise a filter-based normal estimation method using the depth image. Then, we use the curb pattern in the normal image to detect the curb point features row by row. The height property of curbs, or more precisely the fact that curbs are above the road surface from 5 to 35 cm, is also utilized for filtering out the non-road region.

#### 3.2.1. Filter-Based Normal Estimation

In 3D information processing, surface normal direction estimation is of great importance for robotics/ALV to describe objects and understand the scenes. For unorganized 3D points, the statistics-based method is commonly used, which estimates a plane to fit each point and its neighboring points. However, this method is time-consuming for dense data. In contrast, in [22] the authors demonstrated that well organized depth data can make normal estimation fast, and they used *del* operator to estimate the surface normal in the spherical space. In this work, we use *del* operator together with the depth image representation to derive a filter-based normal estimation algorithm for dense depth images.

In particular, for one point with camera coordinates (*x*, *y*, *z*), its image coordinates (*u*, *v*) and depth value *d*(*u*, *v*) can be calculated by Equations (7), (8) and (9):

(7) $$u=\frac{x}{z}\times f_{x}+c_{x}$$

(8) $$v=\frac{y}{z}\times f_{y}+c_{y}$$

(9) d(u,v)=z

In camera coordinates, the normal estimation is formulated as *del* operator [22]:

(10) $$\nabla \equiv \hat{x}\frac{\partial }{\partial x}+ŷ\frac{\partial }{\partial y}+ẑ\frac{\partial }{\partial z}$$

where *x̂*, *ŷ*, *ẑ* correspond to the unit vectors of different axes.

With the chain rule of the derivative, the partial derivatives in Equation (10) can be expanded as:

(11) $$\frac{\partial }{\partial x}=\frac{\partial }{\partial u}\frac{\partial u}{\partial x}+\frac{\partial }{\partial v}\frac{\partial v}{\partial x}+\frac{\partial }{\partial d}\frac{\partial d}{\partial x}$$

(12) $$\frac{\partial }{\partial y}=\frac{\partial }{\partial u}\frac{\partial u}{\partial y}+\frac{\partial }{\partial v}\frac{\partial v}{\partial y}+\frac{\partial }{\partial d}\frac{\partial d}{\partial y}$$

(13) $$\frac{\partial }{\partial z}=\frac{\partial }{\partial u}\frac{\partial u}{\partial z}+\frac{\partial }{\partial v}\frac{\partial v}{\partial z}+\frac{\partial }{\partial d}\frac{\partial d}{\partial z}$$

We apply the above partial derivatives onto Equations (7), (8) and (9), and then substitute the derivative results into Equation (10), which gives:

(14) $$\begin{array}{l} \nabla \equiv \hat{x}(\frac{\partial }{\partial u}\times \frac{f_{x}}{z}+\frac{\partial }{\partial v}\times 0+\frac{\partial }{\partial d}\times 0)+\\ ŷ(\frac{\partial }{\partial u}\times 0+\frac{\partial }{\partial v}\times \frac{f_{y}}{z}+\frac{\partial }{\partial d}\times 0)+\\ ẑ(\frac{\partial }{\partial u}\times \frac{-x}{z^{2}}\times f_{x}+\frac{\partial }{\partial v}\times \frac{-y}{z^{2}}\times f_{y}+\frac{\partial }{\partial d}\times 1) \end{array}$$

This can be written in a compact matrix form:

(15) $$\nabla \equiv \left[\begin{array}{ccc} \hat{x} & ŷ & ẑ \end{array}\right]•\left[\begin{array}{ccc} 1 & 0 & 0\\ 0 & 1 & 0\\ -\frac{x}{z} & -\frac{y}{z} & 1 \end{array}\right]•\left[\begin{array}{c} \frac{\partial }{\partial u}\times \frac{f_{x}}{z}\\ \frac{\partial }{\partial v}\times \frac{f_{y}}{z}\\ \frac{\partial }{\partial d} \end{array}\right]$$

In the above Equation (15), note that $\frac{x}{z}=\frac{u-c_{x}}{f_{x}}$ and $\frac{y}{z}=\frac{v-c_{y}}{f_{y}}$ are fixed for each point in the image with known intrinsic parameters (*f_x_*, *f_y_*, *c_x_*, *c_y_*); thus they can be calculated and saved beforehand to accelerate the computation. $\frac{\partial }{\partial u}d(u,v)$ and $\frac{\partial }{\partial v}d(u,v)$ actually compute the gradients in the u and v directions of the depth image respectively, which correspond to performing two spatial convolutions (details are given in the following), and $\frac{\partial }{\partial d}d(u,v)\equiv 1$.

For suppressing the noises, we first smooth the depth image with a Gaussian kernel (with kernel width *σ_s_*) before normal estimation. Afterwards, we use the Sobel operators, defined in Equations (16) and (17), to efficiently calculate the gradient in a image convolution manner:

(16) $${\text{Sobel}}_{u}=\left[\begin{array}{ccc} -1 & 0 & 1\\ -2 & 0 & 2\\ -1 & 0 & 1 \end{array}\right]/8$$

(17) $${\text{Sobel}}_{v}=\left[\begin{array}{ccc} -1 & -2 & -1\\ 0 & 0 & 0\\ 1 & 2 & 1 \end{array}\right]/8$$

**Figure 8.**
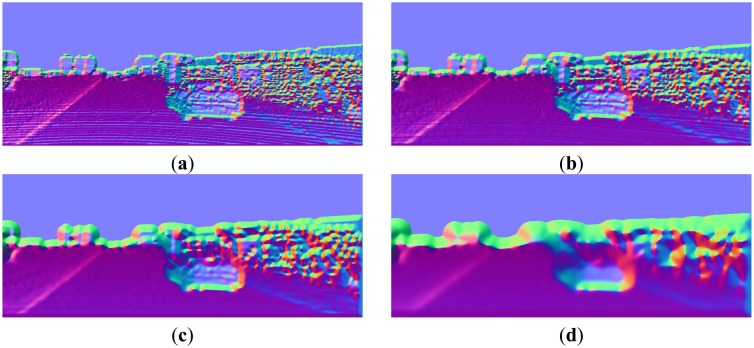
Normal images with different Gaussian kernels: (**a**) *σ_s_* = 1; (**b**) *σ_s_* = 2. (**c**) *σ_s_* = 4; (**d**) *σ_s_* = 8.

The normal estimation results, using the above convolution kernels and different smoothing kernel widths *σ_s_*, are shown in Figure 8. It can be observed that, with appropriate value of *σ_s_*, the convolution method gives pretty good normal estimation results. Generally the best value of *σ_s_* depends on the application, which is a trade-off between reserving the details and suppressing the noises. In this paper, *σ_s_* = 2 is empirically chosen throughout the experiments.

Note that this normal estimation method only needs three spatial convolutions with small kernels and some pixel-level operations, so the computation cost is quite cheap. This method can achieve accurate surface normal estimation for each point in the image. In displaying the normal direction, we use the following color codes throughout the paper. When (*N_x_*, *N_y_*, *N_z_*) is the normal vector of one point, the corresponding pseudo-color used in this paper to show the normal direction is: *r* = (*N_x_* + 1)/2, *g* = (*N_y_* + 1)/2, *b* = (*N_z_* + 1)/2, where (*r*,*g*,*b*) are the red, green, and blue signals respectively.

#### 3.2.2. Curb Point Detection in the Normal Image

**Figure 9.**
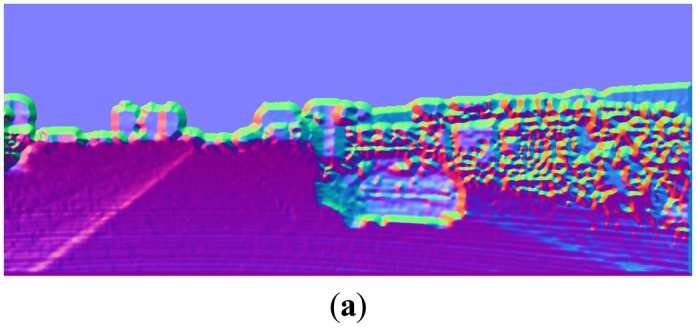

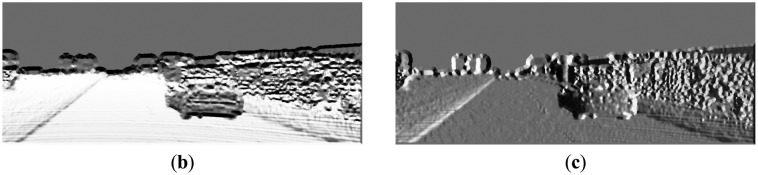
(**a**) Normal image; (**b**) Normal projection in Z_W_; (**c**) Normal projection in X_W_.

As in Figure 9, the normal property of curbs is clearly shown in the normal image and its projections in X_W_ and Z_W_ directions. In particular, in Figure 9b, both sides of the curbs appear a bright-dark-bright (BDB) pattern in row direction. Moreover, in Figure 9c, the left curb appears a dark-bright-dark (DBD) pattern, while the right curb appears a BDB pattern.

**Figure 10.**
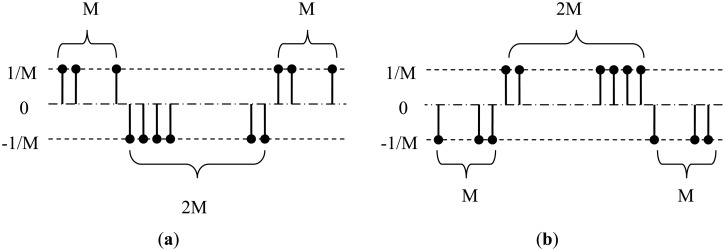
(**a**) ‘bright-dark-bright’ pattern. (**b**) ‘dark-bright-dark’ pattern.

**Figure 11.**
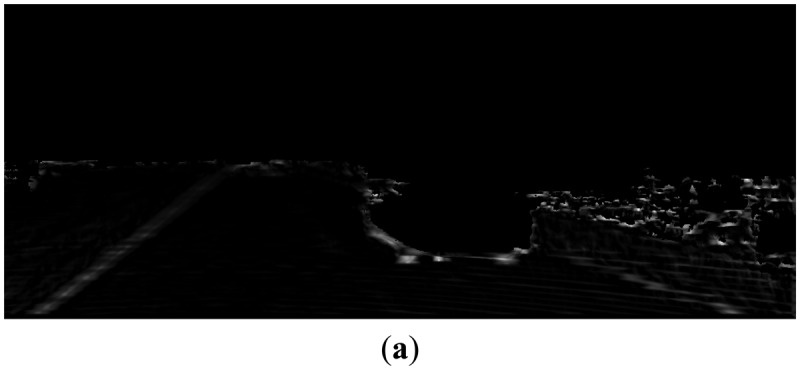

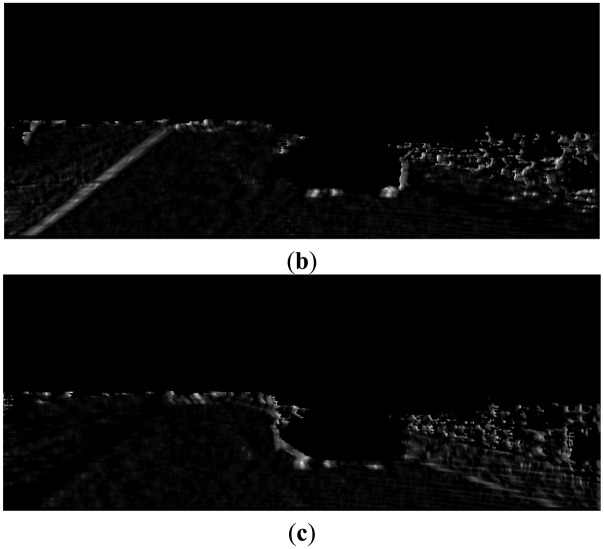
(**a**) ‘BDB’ pattern response on Z_W_ projection map; (**b**) ‘DBD’ pattern response on X_W_ projection map; (**c**) ‘BDB’ pattern response on X_W_ projection map.

Based on above observations, we design multi-scale row patterns for better detecting curb features, which are illustrated in Figure 10. The value of M (in pixel) in Figure 10 controls the detection scale and is chosen from {1, 2, 4, 8, 16} in this paper. For each pattern, the largest response over different scales at one point is taken as its final output at this point. Some examples of the responses of the designed patterns in road region are shown in Figure 11.

**Figure 12.**
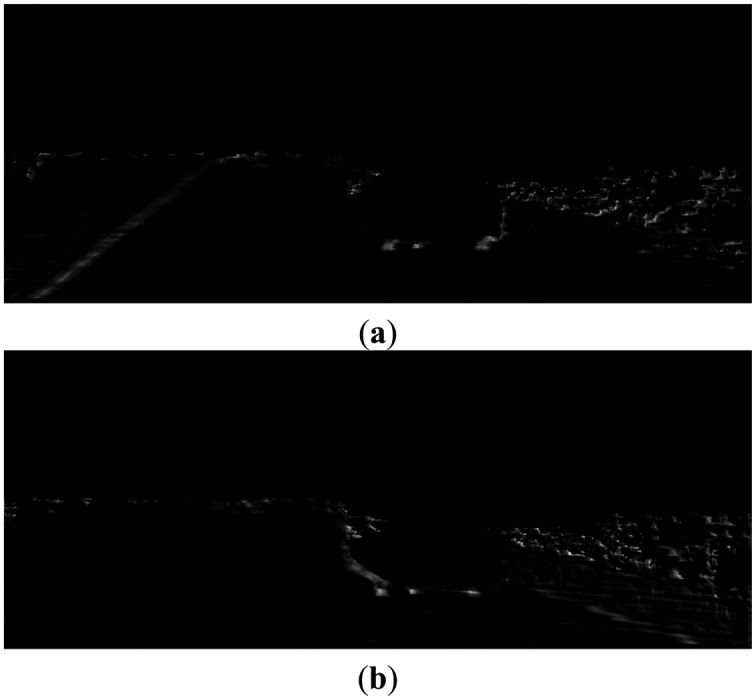
(**a**) Left side curb feature; (**b**) Right side curb feature.

We define the curb feature for each side of the curb based on the pattern responses. The left curb feature, as shown in Figure 12a, is detected by the ‘BDB’ response on the Z_W_ projection map (Figure 11a) multiplied by the ‘DBD’ response on the X_W_ projection map (Figure 11b). Similarly, the right curb feature, as shown in Figure 12b, is detected by the ‘BDB’ response on the Z_W_ projection map (Figure 11a) multiplied by the ‘BDB’ response on the X_W_ projection map (Figure 11c). As observed from the point feature detection results shown in Figure 12, the real curb positions have significant responses, although there are still some isolated noises.

### 3.3. Curb Point Linking

Using the consistency property of the curbs, we build a Markov Chain model for linking the curb points. After transforming the feature responses into node and edge probabilities in the Markov Chain, we can link the best curb path by high efficient dynamic programming algorithm [23,24]. This linking step at the same time filters out the isolated noises and achieves consistent curb paths.

#### 3.3.1. Markov Chain Model for Curb Point Linking

We build a Markov Chain model for the curb in each road side, to make full use of the feature responses and explore the consistency property of the curbs.

Denote the curb point position in each row of the image as a random variable x_i_, and each x_i_ has N different states, each of which corresponds to a specific column of N columns in the image. Here we restrict our model in road region, which is identified by the value z_W_ in Section 3.1.

**Figure 13.**
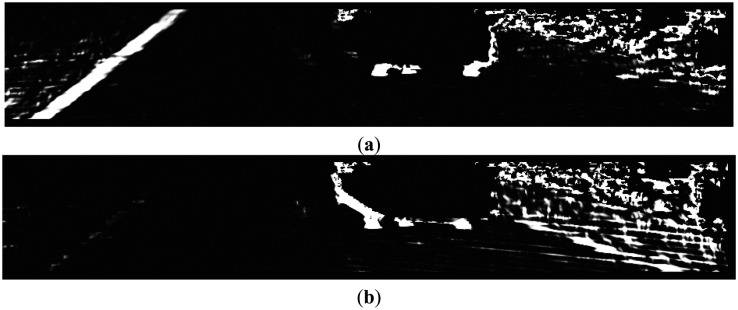
(**a**) Node probability for left side curb; (**b**) Node probability for right side curb.

The node probability (*i.e.*, the probability of xi taking a specific state from the total N states) from the curb feature is defined as:

(18) $$\text{nodePot}(X_{i}^{k})=\frac{1}{Z_{i}}\times (1-\exp(-{\left(\frac{f_{\left(i,k\right)}}{\sigma_{n}}\right)}^{2}))$$

(19) $$Z_{i}=\sum_{k}\left(1-\exp(-{\left(\frac{f_{\left(i,k\right)}}{\sigma_{n}}\right)}^{2})\right)$$

where $X_{i}^{k}$ means that *x_i_* = *k*, namely the curb point in the i-th row locates at k-th column in the image. With this definition of the node probability, we transform the feature response to the probability for the states (column position) of a curb point. The stronger the feature response is, the greater probability the point has. This transforming is controlled by the parameter *σ_n_*, and we set *σ_n_* = 0.003 in all our experiments. Though there are still other ways to define the probability, such as learning-based methods, we prefer this definition for its simplicity of avoiding manually labeling the data. The node probability calculated as above for each side is visualized in Figure 13.

The edge probability which describes the consistency property is defined as:

(20) $$\text{edgePot}(X_{i+1}^{j},X_{i}^{k})=e_{x}(X_{i+1}^{j},X_{i}^{k})\times e_{f}(X_{i+1}^{j},X_{i}^{k})$$

(21) $$e_{x}(X_{i+1}^{j},X_{i}^{k})=\exp(-\frac{{\left(j-k\right)}^{2}}{\sigma_{x}^{2}})$$

(22) $$e_{f}(X_{i+1}^{j},X_{i}^{k})=\exp(-\frac{{\left(f_{\left(i+1,j\right)}-f_{\left(i,k\right)}\right)}^{2}}{\sigma_{f}^{2}})$$

The major consideration in defining this edge probability is that the best path should be smooth in terms of both position and feature. The feature *f* here can be of various types, such as position, texture, color, *etc.* In this paper, we use the curb point feature response as *f* for efficiency, and we choose *σ_f_* = 0.001 and *σ_x_* = 10 in all our experiments.

#### 3.3.2. Link the Curb Points via Dynamic Programming

With the node and edge probabilities defined above, the best path (linking curb points with the largest total probability) can be obtained by applying dynamic programming algorithm. The linking process includes forward and backward searching steps.

In the forward searching steps, the algorithm selects the path from top to bottom. We calculate the probability from the top row (in road region) to current point with Equation (23) and find the best link point (with the largest cumulative probability) with Equation (24):

(23) $$p(X_{i+1}^{j})=\text{nodePot}(X_{i+1}^{j})\times \underset{k\in N(j)}{\max}(p(X_{i}^{k})\times \text{edgePot}(X_{i}^{k},X_{i+1}^{j}))$$

(24) $$\text{link}(X_{i+1}^{j})=\underset{k}{\arg}\underset{k\in N(j)}{\max}(p(X_{i}^{k})\times \text{edgePot}(X_{i}^{k},X_{i+1}^{j}))$$

**Figure 14.**
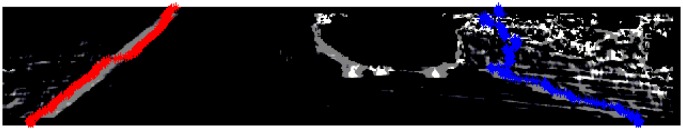
Best path for each side. Red points indicate the left curb, blue points indicate the right curb.

In the backward steps, we choose the point with the maximum probability in the bottom row, and track back to the top by using the link data. In this way, best paths can be linked to filter the isolated noises. The Markov Chain based curb point linking results are shown in Figure 14. We can see that the curb points are linked together accurately. On the right side, some non-curb points are also included because of the occlusion. This motivates us to propose following post-processing steps to filter out these outliers and build the curb models.

### 3.4. Curb Refinement

In this subsection, we introduce the details of employed post-processing in this work for further refining the curb detection results.

#### 3.4.1. Noises Filtering

By analyzing the positions and the curb point features along the best path, we detect suitable break points to cut the best path into several segments, and choose the best segment as our final output to filter out the non-curb parts. After an average smoothing, we calculate the curvature and feature variation along the best path. Throughout the experiments, the break points are defined as points with the curvature greater than 10 or the feature variation greater than 0.003. We sum up the probabilities along each segment, and choose the segment with the largest probability as the output best segment.

#### 3.4.2. Curb Modeling

We use a polynomial model (up to second order) in the curb modeling, which is given in Equation (25). There are also other choices for curb modeling [8–10]. We choose this polynomial one based on its simplicity and its reliability in the experiments:

(25) $$u=a\times v^{2}+b\times v+c$$

We use weighted least square for estimating parameters (a,b,c) by using the nodePot as the weight for each point. For optimizing the parameters, we minimize the objective function in Equation (26):

(26) $$\underset{a,b,c}{\min}\sum_{i}w_{i}\times {\left(u_{i}-a\times v_{i}^{2}-b\times v_{i}-c\right)}^{2}$$

Here (u_i_, v_i_) is a curb point in the best segment, and w_i_ is the $\text{nodePot}(X_{v_{i}}^{u_{i}})$. With a weighted least square method, the best parameters can be estimated by solving Equation (27). The Equation (27) actually has closed form solution and can be solved fast:

(27) $$\left[\begin{array}{c} \underset{i}{\text{∑}}w_{i}\times u_{i}\times v_{i}^{2}\\ \underset{i}{\text{∑}}w_{i}\times u_{i}\times v_{i}^{1}\\ \underset{i}{\text{∑}}w_{i}\times u_{i}\times 1 \end{array}\right]=\left[\begin{array}{ccc} \underset{i}{\text{∑}}w_{i}\times v_{i}^{4} & \underset{i}{\text{∑}}w_{i}\times v_{i}^{3} & \underset{i}{\text{∑}}w_{i}\times v_{i}^{2}\\ \underset{i}{\text{∑}}w_{i}\times v_{i}^{3} & \underset{i}{\text{∑}}w_{i}\times v_{i}^{2} & \underset{i}{\text{∑}}w_{i}\times v_{i}^{1}\\ \underset{i}{\text{∑}}w_{i}\times v_{i}^{2} & \underset{i}{\text{∑}}w_{i}\times v_{i}^{1} & \underset{i}{\text{∑}}w_{i}\times 1 \end{array}\right]•\left[\begin{array}{c} a\\ b\\ c \end{array}\right]$$

#### 3.4.3. Confidence Scoring

We give our confidence scores for the detected curbs based on both the node probability and the accuracy of the model:

(28) $$\text{score}=\underset{i}{\text{∑}}w_{i}\times \exp(-\frac{{\left(u_{i}-a\times v_{i}^{2}-b\times v_{i}-c\right)}^{2}}{\sigma_{sc}^{2}})$$

**Figure 15.**
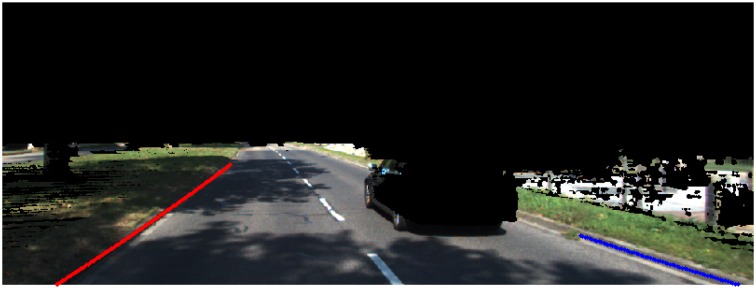
Final results. The confidence score for left curb is 36.6604, and the right is 12.7776.

That is to say, more points lying in the path with stronger curb feature and less model error can lead to a higher score for the detection result. The refined results are shown in Figure 15, where we set *σ_sc_* = 3.

## 4. Experiment Results and Discussion

In this section, we present the experimental evaluations of our proposed method for curb detection. Here we use the widely used KITTI dataset [25] as the test bed for our curb detection method in urban environments. Based on the results in typical scenes, both qualitative and quantitative analyses of the performance of our method are given.

### 4.1. Dataset

We use the KITTI dataset in this section to evaluate our method. The KITTI dataset is one of the most comprehensive datasets for ALV applications, which is commonly used as the test bed for various tasks [25]. In the KITTI dataset, synchronous data from an Inertial Measurement Unit (IMU), a 3D-Lidar (Velodyne Lidar), two stereo cameras are provided at 10 Hz. The camera images are rectified, and the cameras are triggered by the 3D-Lidar to guarantee them to be well aligned. Moreover, the accurate calibration parameters for all sensors are provided.

### 4.2. Curb Detection Results under Various Conditions

We conduct comprehensive evaluations of our method on KITTI dataset, and our proposed method achieves outperforming results. In this section, we present some evaluation results under different conditions.

**Figure 16.**
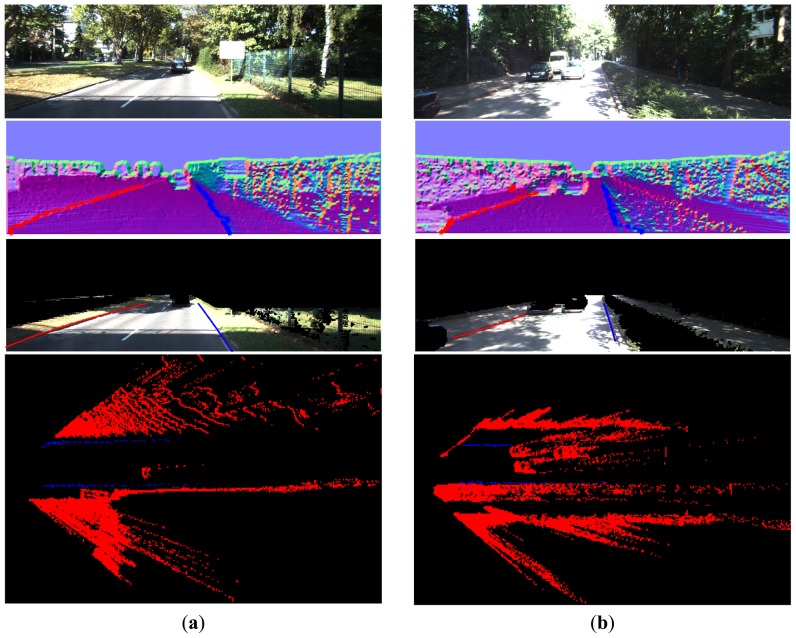
(**a**) Curbs in illumination change; (**b**) Curbs in strong shadow and with obstacle occlusion.

From Figures 16^17^, 18, 19, 20 and 21, the first row shows the visual image, the second row shows the normal image together with the best curb paths, the third row shows road image together with final curb detection results, and the last row shows the result from the top view. In the top view map, red points represent the obstacles, blue points represent the curb results, and the cover range is 60×40 m.

The statistics results of each experiment are summarized in Table 1. For each experiment, we provide the curb detection range in world coordinates and the confidence score for each side.

#### 4.2.1. Illumination Change and Strong Shadow

Figure 16 gives the results in the scenes with illumination change and strong shadow. As can be observed from the results, our method provides results resistant to the shadow influence as it uses the geometric properties for curb detection. In Figure 16a, some curbs without corresponding edges in the image are also reliably detected.

#### 4.2.2. Different Road Widths

**Figure 17.**
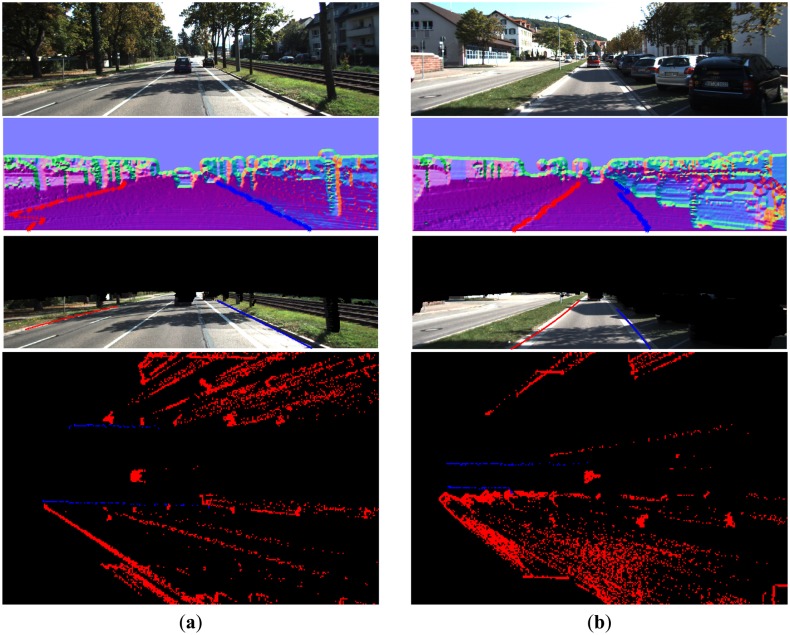
(**a**) Curbs in wide road; (**b**) Curbs in narrow road.

Figure 17 shows the curb results with different road widths. Our method suits for both narrow and wide roads and provides satisfactory results. In Figure 17a, it is shown that our method can detect the left curb which is quite far away. The detection range in horizontal is from −8 to 8 m, and in vertical is from 6 to 30 m, as summarized in Table 1. This detection range is quite sufficient in practice.

#### 4.2.3. Straight and Curved Curbs of Different Lengths

**Figure 18.**
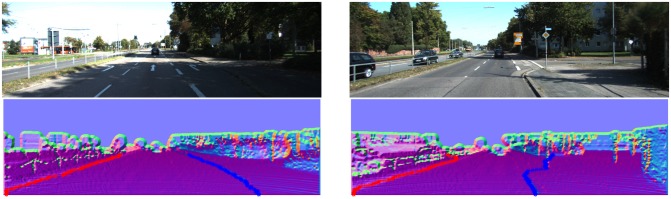

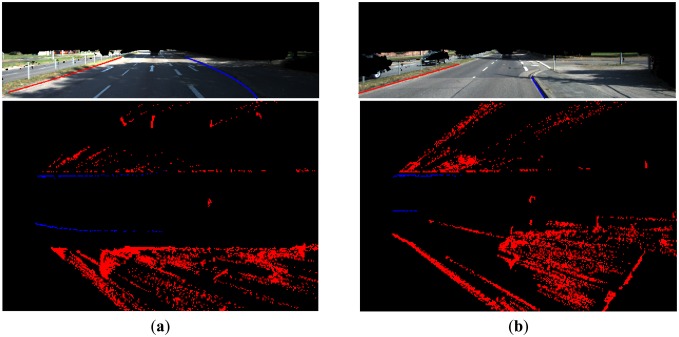
(**a**) Straight and curved curbs; (**b**) Long and short curbs.

For detecting both curved and straight curbs of different lengths, our method provides reliable detection results, as shown in Figure 18. In Figure 18b, the right curb, which only occupies a small part of the road, is also successfully detected, through our proposed linking and post-processing steps.

#### 4.2.4. Obstacle Occlusion

**Figure 19.**
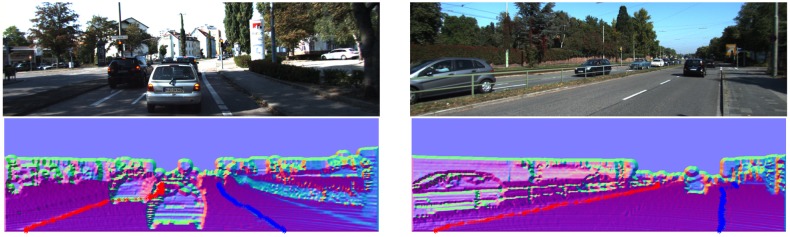

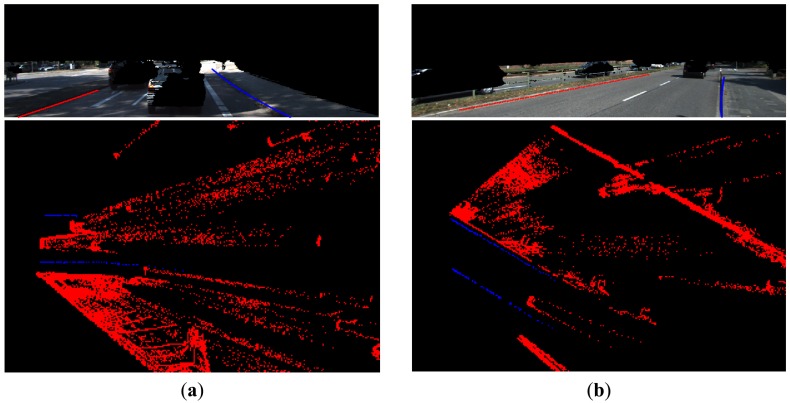
(**a**) Curbs with obstacle occlusion; (**b**) Vehicle direction change situation.

Figure 19a and Figure 16b give the curb detection results with obstacle occlusion. In Figure 19a, though there are objects aside and ahead, our method can still provide reasonable results. Because our method uses the height property of curbs and only detects curbs in the identified road region, the obstacle region can be filtered out. This makes our method robust to the obstacle occlusion.

#### 4.2.5. Vehicle Direction Change

Figure 19b gives the curb detection result in the scene where the vehicle direction changes. In this experiment, the direction of the vehicle relative to the road is changed to 30–45 degrees. It clearly shows that our method still outputs reliable detection results.

#### 4.2.6. Broken Curbs

**Figure 20.**
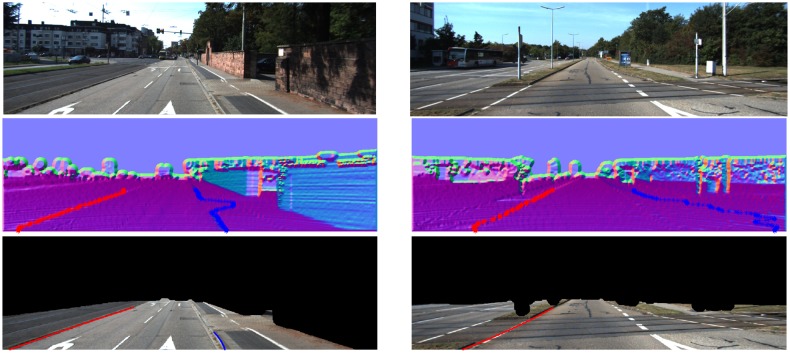

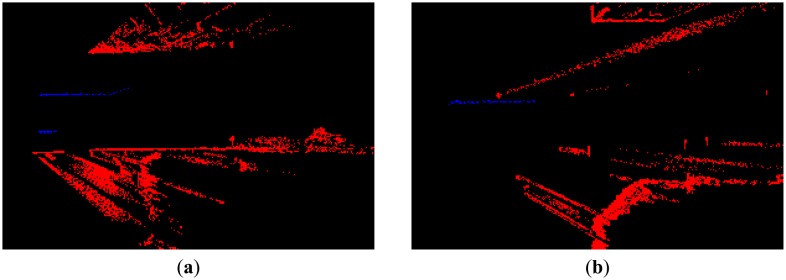
(**a**) Partly detected broken curbs; (**b**) Completely detected broken curbs.

For broken curbs, our method can provide reasonable results, as shown in Figure 20. Because we use break points to filter the noises, our method generally cannot link the broken curbs and will output the strongest segment, as the right curb in Figure 20a. However, if the broken part has consistent feature (even very weak), our method can completely detect and link the broken curbs, as the left curb in Figure 20b.

#### 4.2.7. Missed Curbs

**Figure 21.**
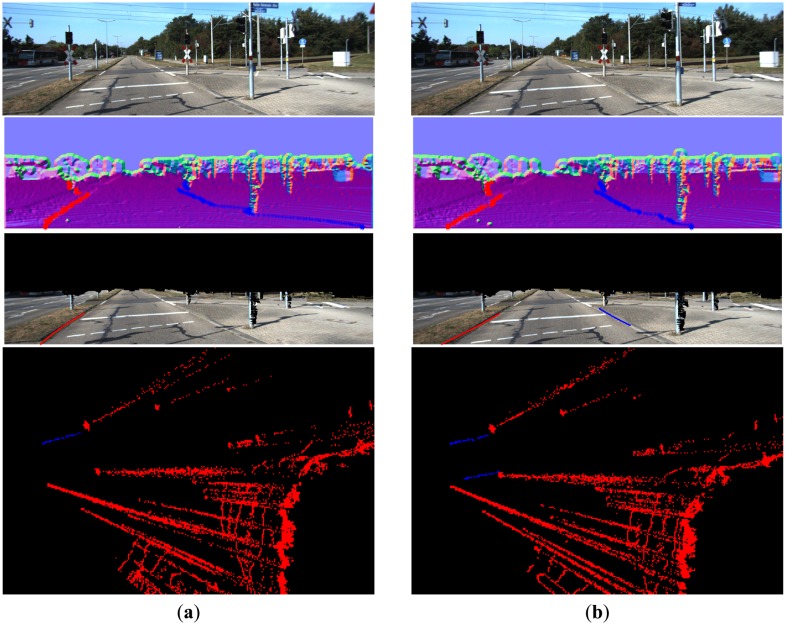
(**a**) Missed curbs; (**b**) Detected curbs.

With confidence scores, our method can judge whether there is a curb in each side. In our experiments, if the confidence score is lower than 10, we disable the result. Figure 21 gives the missed curb results in sequential frames. In Figure 21a, the right curb is weak and cannot be reliably detected. In the next frame, when the curb features become stronger, we can detect the right curb, as shown in Figure 21b.

### 4.3. Quantitative and Qualitative Analyses on Our Method

In this subsection, we provide some further discussions and illustrations on our proposed method.

#### 4.3.1. Edge Probability Design

In the edge probability design, we use both the position and the feature. In this subsection, we give a comparison with different probability strategies. One is our method and the other one only uses the position information.

**Figure 22.**
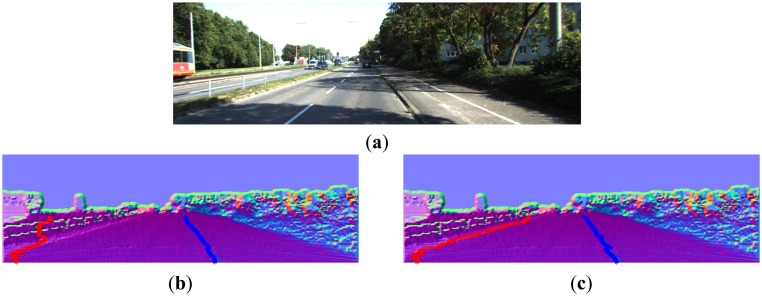
(**a**) Visual image. (**b**) The linking results with only position information. (**c**) Our linking results.

Figure 22 shows a demonstration result: (a) is the visual image of the scene; (b) is the output with only position information; (c) shows our best curb linking results. Because our method includes the curb feature, it successfully tracks the consistent curb points, and filters out isolated noises. Thus our method finally provides better results than the baseline method.

#### 4.3.2. Used Computation Resource

In each step of our method, we take the implementation efficiency into account. The depth recovery and normal estimation take the major part of the computation resource in our method. For depth recovery, we use the Graphic Processing Unit (GPU) implementation from [4], and the processing time for each frame in KITTI is about 30 ms. For normal estimation with depth images, we derive a filter-based method, which only needs some small-size convolutions and pixel-level operations, and the processing time with Central Processing Unit (CPU) is about 15 ms in our experiments. Finally, we achieve 15 Hz (15 frames per second) by using an i7 processor together with a desktop graphics card (NVIDIA GTX260). The total implementation time for each frame in KITTI dataset is about 60 ms, which achieves real-time performance.

#### 4.3.3. The Detection Range

One of the most important advantages of our method is its robustness. By using the dense depth image, our method achieves reliable results even for quite noisy scenes. Our method also achieves larger detection range. In KITTI dataset, in no occlusion condition, the detection range is about +30 m in vertical and +8 m in horizontal for common curbs with about 10 cm height. The typical results are shown in Figures 16a, 17a, 18a, and the statistics results are listed in Table 1.

## 5. Conclusions and Future Work

In this paper, we have proposed a curb detection method based on fusing the 3D-Lidar and camera data. Using the dense depth image from range-visual fusion, we derived a filter-based method for efficient surface normal estimation. By using the specifically designed pattern of curbs, curb point features were detected in the normal image row by row. We then formulated the curb point linking process as a best path searching on a corresponding Markov Chain, which was solved via dynamic programming. We also designed several post-processing steps to filter the noises, parameterize the curb models and compute their confidence scores. Comprehensive evaluations on KITTI dataset showed that our method achieved good results in both static and dynamic scenes, and processed the data at the speed of 15 Hz. For the obstacle occlusion and strong shadow, our method showed strong robustness. In typical scenes without occlusion, our detection rang reached 30 m for front and 8 m for each side.

In the future, we are going to apply this curb detection method for other ALV applications, such as map building, vehicle localization and so on. To date, there is no widely accepted benchmark for curb detection. A comprehensive benchmark with different sensors for curb detection is needed for fair comparison, and this could be our future work.

## Figures and Tables

**Table 1.** Detection range and confidence scores of each experiment.

**Experiments**	**Sides**	**Detection Range (meters)**				**Scores**

		**Min(X_W_)**	**Max(X_W_)**	**Min(Y_W_)**	**Max(Y_W_)**	
Figure 16a	Left	−6.19	−4.99	6.08	27.58	24.93
	Right	0.99	1.34	6.26	**30.63**	30.30
Figure 16b	Left	−5.47	−5.28	8.05	15.63	19.58
	Right	0.47	0.79	7.43	**30.19**	62.14
Figure 17a	Left	**−8.41**	−7.89	10.88	25.04	12.89
	Right	3.65	4.72	6.52	**32.93**	42.16
Figure 17b	Left	−2.33	−2.01	**5.86**	28.14	38.60
	Right	1.42	2.47	6.06	16.16	18.08
Figure18a	Left	−6.55	−5.17	6.88	**30.78**	46.43
	Right	3.43	5.06	6.31	**30.30**	46.24
Figure 18b	Left	−5.84	−5.39	6.50	19.28	26.17
	Right	0.97	1.11	6.38	10.71	36.69
Figure 19a	Left	−4.90	−4.67	6.51	11.53	33.47
	Right	2.51	4.07	**5.72**	28.63	24.47
Figure 19b	Left	−4.11	5.56	6.48	22.98	28.39
	Right	3.76	**13.21**	6.60	22.62	63.25
Figure 20a	Left	−5.92	−4.77	6.19	20.48	43.45
	Right	0.99	1.13	**5.94**	8.79	17.93
Figure 20b	Left	−4.00	−3.45	6.23	20.06	32.06
	Right	-	-	-	-	0
Figure 21a	Left	−5.98	−4.27	6.55	12.68	25.76
	Right	-	-	-	-	0
Figure 21b	Left	−5.63	−4.29	6.31	12.48	29.11
	Right	0.24	1.43	8.70	14.14	21.30
